# Characterization of the human aqueous humour proteome: A comparison of the genders

**DOI:** 10.1371/journal.pone.0172481

**Published:** 2017-03-08

**Authors:** Natarajan Perumal, Caroline Manicam, Matthias Steinicke, Sebastian Funke, Norbert Pfeiffer, Franz H. Grus

**Affiliations:** Experimental and Translational Ophthalmology, Department of Ophthalmology, University Medical Center of the Johannes Gutenberg University Mainz, Mainz, Germany; Pacific Northwest National Laboratory, UNITED STATES

## Abstract

Aqueous humour (AH) is an important biologic fluid that maintains normal intraocular pressure and contains proteins that regulate the homeostasis of ocular tissues. Any alterations in the protein compositions are correlated to the pathogenesis of various ocular disorders. In recent years, gender-based medicine has emerged as an important research focus considering the prevalence of certain diseases, which are higher in a particular sex. Nevertheless, the inter-gender variations in the AH proteome are unknown. Therefore, this study endeavoured to characterize the AH proteome to assess the differences between genders. Thirty AH samples of patients who underwent cataract surgery were categorized according to their gender. Label-free quantitative discovery mass spectrometry-based proteomics strategy was employed to characterize the AH proteome. A total of 147 proteins were identified with a false discovery rate of less than 1% and only the top 10 major AH proteins make up almost 90% of the total identified proteins. A large number of proteins identified were correlated to defence, immune and inflammatory mechanisms, and response to wounding. Four proteins were found to be differentially abundant between the genders, comprising SERPINF1, SERPINA3, SERPING1 and PTGDS. The findings emerging from our study provide the first insight into the gender-based proteome differences in the AH and also highlight the importance in considering potential sex-dependent changes in the proteome of ocular pathologies in future studies employing the AH.

## Introduction

Proteomic studies of the aqueous humour (AH) has been a subject of much interest owing to the multiple physiological roles of the AH in maintaining proper functionality of the ocular system. The human AH is secreted and regulated in the immunopriviledged compartments of the eye, and therefore, is composed of a complex mixture of proteins, solutes, growth factors and electrolytes that supply nutrients to the avascular tissues in the eye [1, 2]. In addition, the AH also plays important functions in removal of excretory metabolic products from ocular tissues and has active participation in the immune response against invading pathogens and inflammation [1–5]. The characterization of the human AH proteome saw massive progress in the last decade, especially instrumental in defining specific changes associated with the pathogenesis of myriad eye diseases, such as primary open angle glaucoma (POAG) [6–8], primary congenital glaucoma [9], cataract [1, 2, 5, 10], myopia [11], Coats' disease [12], glaucoma with pseudoexfoliation syndrome [13], keratoconus [14], branch retinal vein occlusion (BRVO) [15], acute corneal rejection [16] and age-related macular degeneration (AMD) [17, 18]. However, the gender-specific differences in AH proteome are yet to be explored. Much attention has been directed towards gender-based medicine in recent years because the prevalence of many well-characterized ocular pathologies are significantly skewed towards a particular sex [19–25]. It has also been reported that there are substantial variations in the blood plasma proteome of males and females [26]. Since the AH is an ultrafiltration of blood, we hypothesize that there may also be gender-specific differences in the AH proteome.

On the other hand, among the most abundant proteins found in the AH, albumin (ALB), immunoglobulin G (IgG), immunoglobulin A (IgA), transferrin, haptoglobin and antitrypsin represent the major ones. It is well-recognized that one or more of these proteins are generally depleted in the AH samples prior to proteomics analysis to reduce the masking effect of the abundant proteins [1, 2, 10, 17, 27–29]. Nevertheless, the removal of these abundant proteins is associated with several drawbacks, mainly in the loss of information of interacting proteins. In retrospect, a study by Granger *et al* demonstrated that the depletion of ALB, which is a known carrier protein, resulted in a loss of cytokines [30]. Besides, depletion steps will also result in the loss of crucial information of specific proteins by non-specific binding either with the matrix or the bait of the antibody that may occur during affinity purifications [31]. Hence, discovery proteomics strategy without depletion steps will enable to retain the abundant proteins together with their interacting partners in the AH proteome. This strategy is also important for comprehensive proteomic analyses for better understanding of the protein-protein interactions and potential alterations attributable to specific ocular disease conditions involving these abundant proteins.

Therefore, this study was undertaken to assess the differences between sexes in the AH proteome employing the mass-spectrometry (MS)-based proteomic strategy sans the depletion steps.

## Materials and methods

### Sampling

This study was performed in strict adherence to the guidelines of the 1964 Declaration of Helsinki and all experimental protocols of this study were approved by the “Landesärztekammer Rheinland-Pfalz” ethics committee. All participants were informed of the possible risks, the goal of the study and privacy policy, and an informed consent was signed according to the recommendations of the Declaration of Helsinki for investigation with human subjects. In this study, AH from 30 patients who underwent cataract surgery was utilized. The samples were stored immediately at -80°C after collection, prior to analysis. The AH samples were equally divided to male (N = 15) and female (N = 15) groups, age 70 ± 7 and 73 ± 9, respectively. The samples were further divided into three biological replicates comprising 5 samples in each group, designated as Male_R1 (N = 5), Male_R2 (N = 5), Male_R3, Female_R1 (N = 5), Female_R1 (N = 5) und Female_R1 (N = 5) for the discovery proteomics analysis. The total protein concentrations in the collected AH samples were determined using BCA Protein Assay Kit (Pierce, Rockford, IL). Table A in S1 File summarizes the general description of the study samples and the total protein concentration yielded from each individual.

### Label-Free Quantification (LFQ) analysis

Label-free quantification of peptides *via* one-dimensional electrophoresis (1DE) and liquid chromatography (LC)—electrospray ionization (ESI)-MS/MS strategy was employed to identify the change in protein abundance in the designated groups. The AH samples for each assigned group (N = 5) were pooled equally (18 μg per individual) to a total of 90 μg with three biological replicates. The pooled AH samples were subjected to 1DE (30 μg/ well with total of three wells per group), using 4–12% Bis-Tris Gels (Invitrogen, Karlsruhe, Germany) with MES running buffer under reducing conditions for 60 minutes with a constant voltage of 150 V. The gels were stained with Colloidal Blue Staining Kit (Invitrogen) according to the manufacturer's instructions. The visualized protein bands were sliced into 12 gel pieces for each biological groups and were subjected to trypsin digestion and the peptides were extracted utilizing methods previously described [32].

The LC-system consisted of a Rheos Allegro pump (Thermo Scientific, Rockford, USA) and a PAL HTC autosampler (CTC Analytics, Zwingen, Switzerland), as described elsewhere [33]. The system comprised of a 30 × 0.5 mm BioBasic C18 precolumn (Thermo Scientific) connected to a 150 × 0.5 mm BioBasic C18 column (Thermo Scientific). Solvent A was LC-MS grade water with 0.1% (v/v) formic acid, and solvent B was LC-MS grade acetonitrile with 0.1% (v/v) formic acid. The gradient was run for 90 min per gel spot as follows; 0–50 min: 10–35% B, 50–70 min: 35–55% B, 70–75 min: 55–90% B, 75–80 min: 90% B, 80–83 min: 90–10% B und 83–90 min: 10% B. Continuum mass spectra data were acquired on an ESI-LTQ-Orbitrap-XL MS (Thermo Scientific, Bremen, Germany). The LTQ-Orbitrap was operated in a data-dependent mode of acquisition to automatically switch between Orbitrap-MS and LTQ-MS/MS acquisition. Survey full scan MS spectra (from *m*/*z* 300 to 2000) were acquired in the Orbitrap with a resolution of 30000 at *m/z* 400 and a target automatic gain control (AGC) setting of 1.0 × 10^6^ ions. The lock mass option was enabled in MS mode and the polydimethylcyclosiloxane (PCM) *m/z* 445.120025 ions were used for internal recalibration in real time [34]. The five most intense precursor ions were sequentially isolated for fragmentation in the LTQ with a collision-induced dissociation (CID) fragmentation, the normalized collision energy (NCE) was set to 35% with activation time of 30 ms with repeat count of 3 and dynamic exclusion duration of 600 s. The resulting fragment ions were recorded in the LTQ.

The acquired continuum MS spectra were analysed by MaxQuant computational proteomics platform version 1.4.1.2 and its built-in Andromeda search engine for peptide and protein identification, with LFQ and Intensity-based absolute quantification (iBAQ) algorithm enabled [35–39]. The tandem MS spectra were searched against Uniprot Human database (date, 12.01.2015) using standard settings with peptide mass tolerance of ± 30 ppm, fragment mass tolerance of ± 0.5 Da, FDR for peptide and protein identification was set to 0.01 with ≥ 6 amino acid residues and only “unique plus razor peptides” that belong to a protein were chosen to be included for quantification [35]. Carbamidomethylation of cysteine was set as a fixed modification, while protein N-terminal acetylation and oxidation of methionine were defined as variable modifications, enzyme: trypsin and maximum number of missed cleavages: 2. The output of the generated “proteingroups.txt” data from the MaxQuant analysis was utilized for Pearson correlation and statistical analysis using Perseus software [40]. For statistical analysis, two-samples Student t-test-based statistics with P < 0.02 was applied on Log2 transformed LFQ values and the minimum number of values “in at least one group” is 3 to assert proteins regulation as significant for the specific groups. Box plots of the differentially abundant AH proteins were plotted using Statistica (v8, StatSoft, Tulsa, OK).

### Functional annotation and pathways analysis

Proteins determined to be differentially abundant were tabulated in Excel and their gene names were used for functional annotation and pathways analysis. First, DAVID tool (version 6.7) (http://david.abcc.ncifcrf.gov/home.jsp) was used for interpreting the gene ontology biological process (GOBP) terms of the identified AH proteins, as described elsewhere [41, 42]. The Ingenuity Pathways Analysis software (IPA, Ingenuity QIAGEN Redwood City, CA) (www.qiagen.com/ingenuity) was used for interpreting the gene ontology cellular component (GOCC), molecule types and protein-protein interaction (PPI) networks of the identified AH proteins, as described elsewhere [42].

## Results

**Fig 1:**
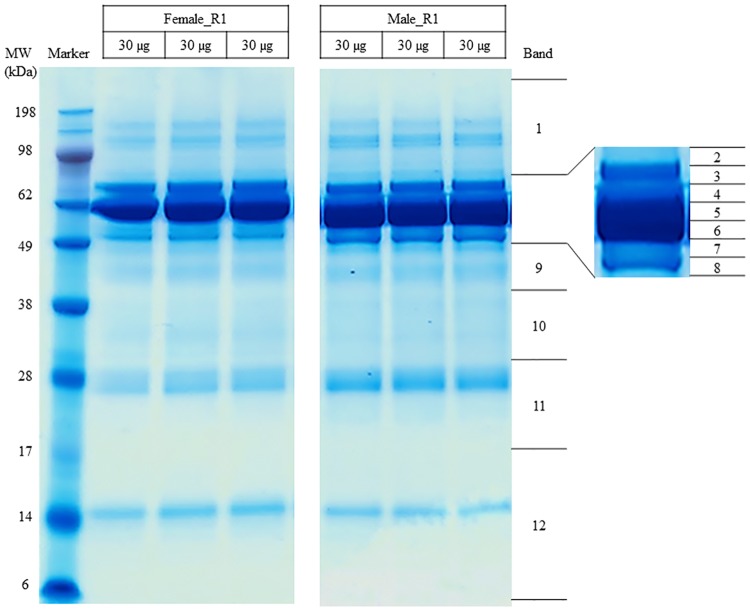
Representative AH protein profiles of the female and male groups resolved in 1DE gel after colloidal blue staining.

**Fig 2:**
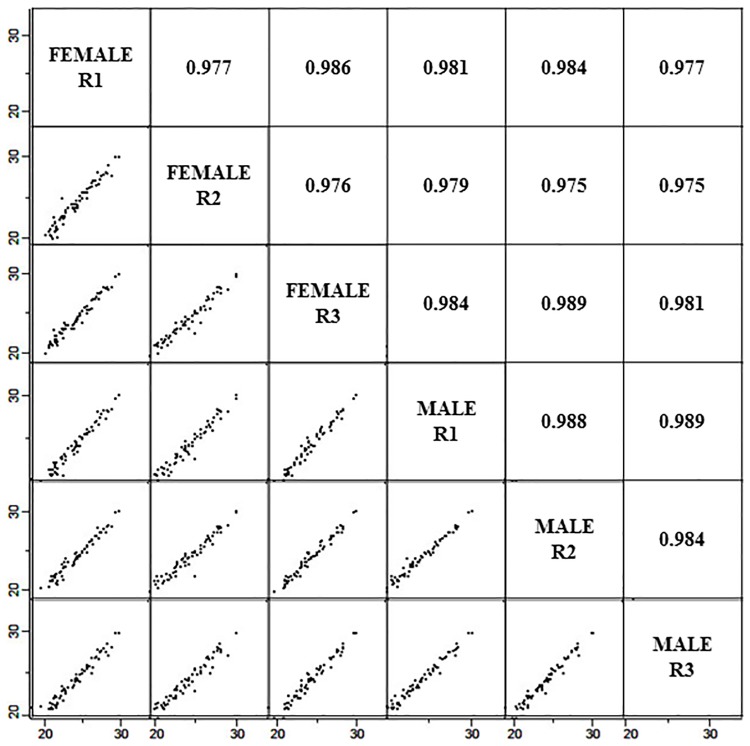
The degree of proteome variances of the female and male groups investigated by Pearson correlation analysis.

**Fig 3:**
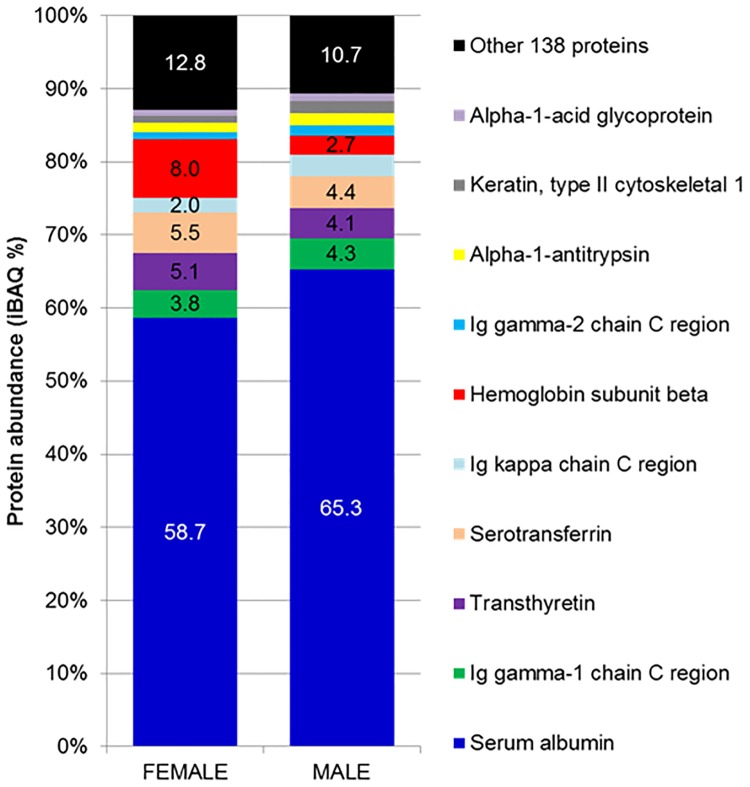
The bar chart shows the degree of protein abundance in the female and male groups.

The representative AH protein profiles of males and females resolved in 1DE gel are illustrated in Fig 1. Since these experiments were performed without any depletion steps, several thick protein bands were visualized in the molecular weight region of 49 to 70 kDa that represent albumin. This region was sliced into 7 different bands for subsequent analysis with the aim to reduce the masking effect of the abundant proteins during the MS analysis. We were also careful not to overload this protein band region during the 1DE, by utilizing only 30 μg proteins for each well and three wells were pooled based on the designated region, as represented in Fig 1. This finally resulted in a total of 90 μg proteins per biological replicate. A total of 147 proteins were identified by the discovery approach and the details of these proteins are as presented in Table B in S1 File. On average, the Pearson correlation between female and male groups was 0.981 ± 0.005, as shown in Fig 2; demonstrating high similarities between the AH proteome of male and female groups. iBAQ analysis demonstrated that only top 10 abundant proteins from the total of 147 proteins identified make up 88.25 ± 2.22% of the total abundance in both groups, as shown in Fig 3 (complete data in Table B in S1 File). Moreover, 61.98 ± 5.78% of the total abundance of the AH proteome comprises albumin.

**Fig 4:**
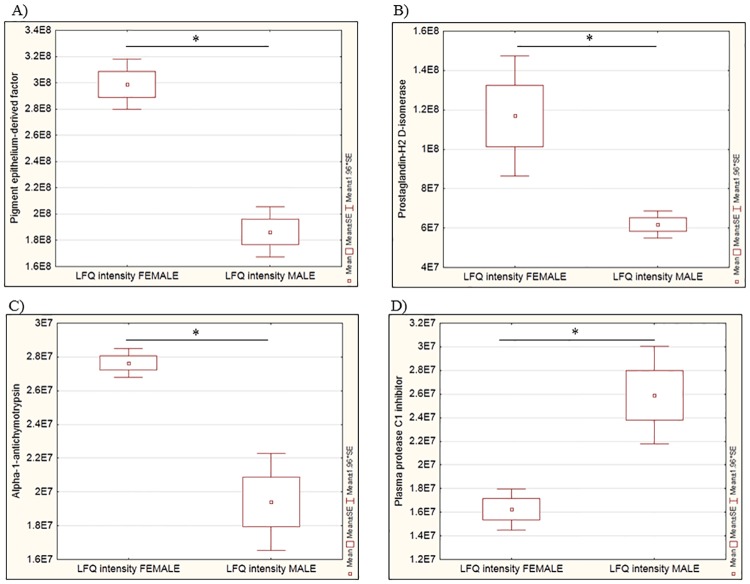
Box plots show the differentially abundant AH protein profiles in the female and male groups employing the discovery proteomics strategy. A) SERPINF1, B) PTGDS, C) SERPINA3 and D) SERPING1. The y-*axis* represents the mean LFQ intensities of the proteins from three biological replicates. Boxes represent the ME ± SE, rectangles the ME ± SD. *p<0.05.

Next, with the use of stringent filtering criteria, the LFQ analysis identified four proteins to be differentially abundant between the genders (complete data in Table C in S1 File). These comprise pigment epithelium-derived factor (SERPINF1), alpha-1-antichymotrypsin (SERPINA3), plasma protease C1 inhibitor (SERPING1) and prostaglandin-H2 D-isomerase (PTGDS), as illustrated in Fig 4. Interestingly, among these four differentially abundant proteins, only SERPING1 was found to be significantly low in abundance in the AH of females in comparison to their male counterparts, whereas all other three differentially abundant proteins were found to be in higher abundance in the female AH proteome.

**Fig 5:**
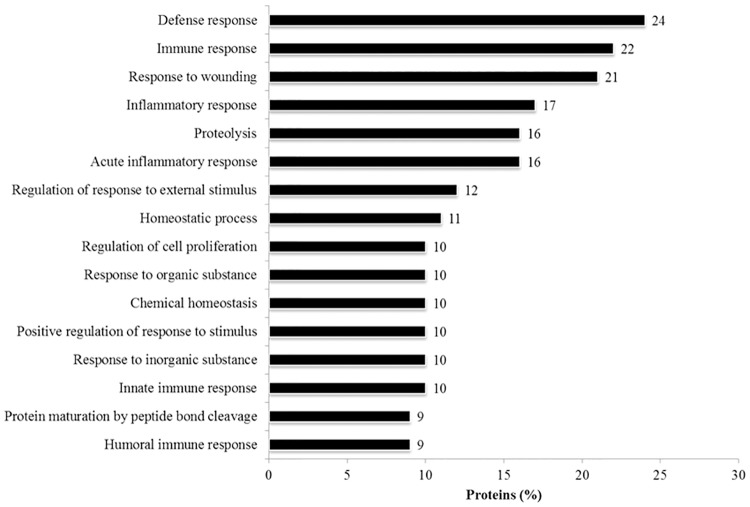
The bar charts shows the over-represented GOBP terms associated with the identified AH proteins analysed employing the DAVID tool.

**Fig 6:**
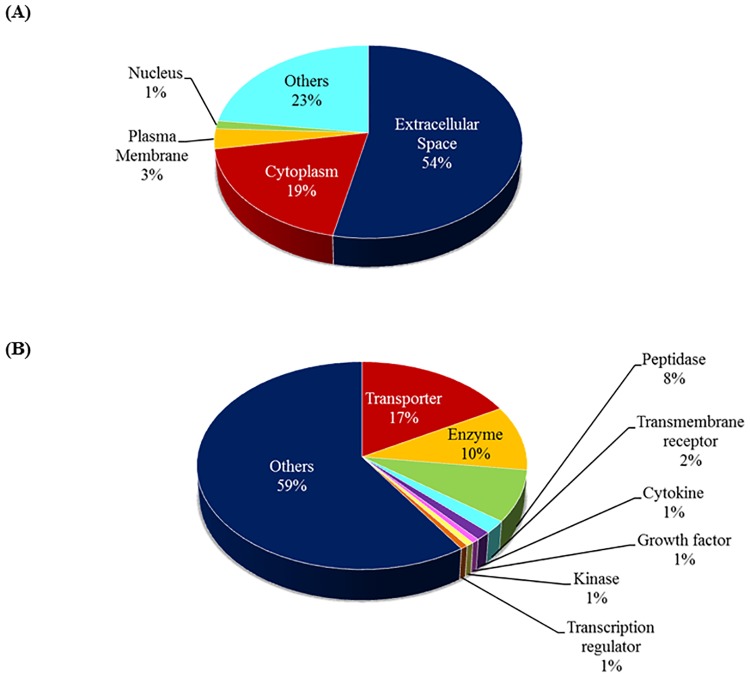
The pie charts shows the over-represented (a) GOCC terms and (b) molecular type terms associated with the identified AH proteins employing the Ingenuity Pathways Analysis software.

**Fig 7:**
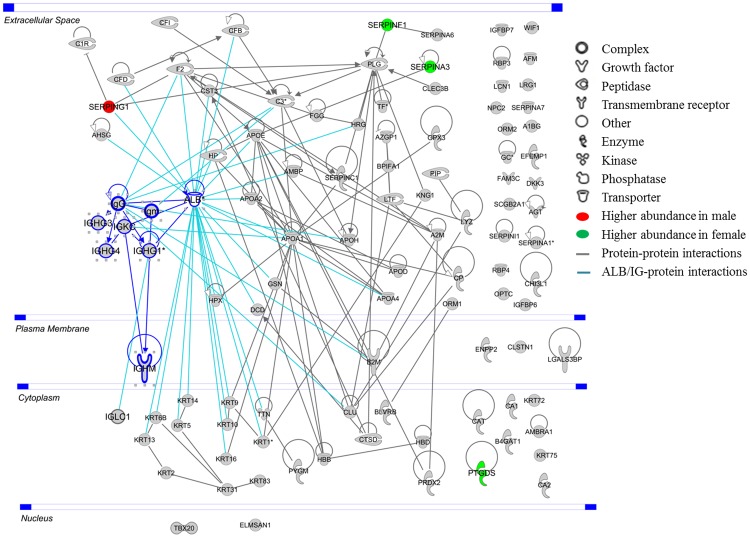
Networks of PPI of the identified AH proteins analysed employing the Ingenuity Pathways Analysis software.

Several categories of major biological processes were observed to be associated with the identified proteins, with the top five representing the defence (24%) and immune responses (22%), response to wounding (21%), inflammatory (17%), and acute inflammatory responses (16%). Additionally, proteolysis (16%), regulation of response to external stimulus (12%) and homeostatic process (11%) are among the other biological mechanisms found to be associated with the AH proteins, as depicted in Fig 5. The complete list of the GOBP of AH proteins identified in this study is in Table D in S1 File. The characteristics of the identified AH proteins were further analysed, and the localization of these proteins in the cell was determined employing the over-represented GOCC terms. A majority of the AH proteins are localized in the extracellular space (54%), while 19% represents cytoplasmic proteins, as shown in Fig 6a. Only a small percentage of membrane-based and nucleus proteins were identified from the human AH, which represents 3% and 1% of the total proteome, respectively. The rest of the proteins were localized in other cellular compartments and these comprises 23% of the AH proteome. The molecular characteristics analysis of the identified AH proteins showed that while a large majority of the proteome is found to possess other functions at the molecular level (59%), many of these proteins are transporters (17%), enzymes (10%) and peptidases (8%). On the other hand, a small percentage of these are categorized as transmembrane receptors (2%), cytokines (1%), growth factors (1%) and kinases (1%), as shown in Fig 6b. The summary of the PPI networks of the identified AH proteins were generated according to their respective GOCC terms and molecule types employing IPA, as depicted in Fig 7 (the complete lists of PPI networks for each comparison are reported in Table E in S1 File). The AH protein interaction network analysis demonstrated that ALB (30 PPI) and immunoglobulins (27 PPI) have interactions and/ or forms complexes with approximately 75% of the total AH proteins, including with three of the differentially abundant proteins, which are SERPINF1, SERPINA3 and SERPING1 (Fig 6).

## Discussion

The past decade has witnessed a paradigm shift in the field of ‘omics’ research with the dawn of the post-genomic proteomic era that has revolutionized biological and clinical investigations. In addition to the facilitation of in-depth analysis of cellular and tissue-based samples, proteomics has emerged as a powerful tool to define the protein ensembles present in body fluids, namely plasma, serum, cerebrospinal fluid, saliva and urine [43–47]. Likewise in ophthalmic research, proteomic analysis is a valuable method that is being extensively used to study the molecular changes in the ocular fluids in physiological and pathological conditions [48–50]. This study endeavoured to profile the inter-gender differences in the proteome of human AH proteins employing the discovery proteomics strategy. The results emerging from the current investigation have demonstrated the existence of specific gender- based variations in the protein profiles of human AH. The use of AH of patients undergoing routine cataract surgery in the current study represents normal control samples in accordance with previous studies, as it is well- recognized that AH samples of healthy individuals cannot be obtained due to ethical reasons [1, 2, 5, 10].

The hallmark of this study is in the identification of four differentially abundant proteins between the genders, which has never been reported hitherto. Interestingly, three of these belong to the serpin superfamily. Considering the ubiquity of the serpin proteins in the human system and their functional relevance in the pathogenesis of many ‘serpinopathies’ when deficient, it is therefore no surprise that previous studies of the human AH have shown significant decrement of SERPINF1 in patients with high myopia [51]. Additionally, decrement of this protein in the ocular system is also associated with neovascularization [52]. This is in agreement with the inherent functions of this 50 kDa secretory glycoprotein, which possess potent anti-angiogenic, anti-thrombotic, antitumorigenic, anti-inflammatory and neuroprotective properties [51–58]. Correspondingly, SERPINF1 has been tested as a potential anti-angiogenic agent, especially in the eye, whereby its protective effects against neovascularization are essential in the AH to maintain the transparency of the cornea and lens [52, 59–61]. Conversely, increased levels of this protein were demonstrated in the aqueous and vitreous humour of AMD patients [17, 62]. Although no gender-based alterations of this protein have been reported in the eye, SERPINF1 level was elevated in serum samples of women with polycystic ovary syndrome associated with insulin resistance and reduced in peritoneal fluid of women with endometriosis [63, 64]. Albeit no specific roles have previously been postulated for SERPINF1 depending on the gender, it is noteworthy that the findings associated with gynaecological diseases are correlated to alterations in the control of physiological angiogenesis.

SERPINA3, which is also part of the serpin family, was also demonstrated to be highly abundant in the female AH samples compared to the male samples. The major role of this protein is to inhibit proteases and thereby, confer protection to tissues from proteolytic damage inflicted by these enzymes [65]. This is an acute phase protein that is induced during inflammation and implicated in several devastating neurological disorders, namely in Alzheimer's and Parkinson's disease, stroke, cerebral haemorrhage and chronic obstructive pulmonary disease [65–68]. Furthermore its expression regulation is increased when the trabecular meshwork is treated with Dexamethasone, an artificial, anti-inflammatory glucocorticoid [69].

The third differentially abundant protein with higher abundance in females in this study was PTGDS. PTGDS catalyses the conversion of prostaglandin H2 (PGH2) to prostaglandin D2 (PGD2) and the levels of this enzyme and its metabolites are greatly elevated during inflammation [70, 71]. Many studies have reported on the expressions levels of PTGDS in various ocular disorders. For example, in retrospect, a study reported a significant increment in the expression level of the enzyme PTGDS in the AH of POAG patients [72]. On the contrary, decreased levels of this enzyme were discovered in samples of congenital glaucoma and keratoconus patients [9, 14]. Although the exact function of this enzyme in the ocular tissue is still unclear, it is presumed to be part of the intraocular pressure maintenance on the one hand and the development and perpetuation of the blood-aqueous-barrier on the other hand [73, 74].

The differentially abundant protein that was found to be significantly decreased in the female AH samples was SERPING1. This is a glycoprotein that belongs to the largest member of the superfamily of serine protease inhibitors, which controls the activation of the activated first component of complement (C1)-complex and also play crucial functions in suppressing inflammatory reactions, fibrinolysis and blood coagulation [75–77]. The regulation profile of this protein was found to be differentially abundant in serum samples of type 1 and 2 diabetes patients, whereas, it is over-expressed in the AH of patients suffering from keratoconus [14, 78].

In this study, three of the differentially abundant proteins of SERPINF1, SERPINA3, SERPING1 were found to have interactions and/ or forms complexes with high abundant AH proteins, especially ALB and immunoglobulins. Generally, these most abundant proteins present in the AH are usually subjected to depletion prior to proteomics analysis to enhance the detection of low abundance proteins to map the protein fingerprints in healthy (cataract) samples and also to find the alterations associated with particular ocular pathologies. [1, 2, 10, 17, 79]. The AH protein-protein interactions analysis from this study provide added evidence that the removal of these abundant proteins might change the composition of interacting protein partners that are bound to them and thereby, alter the innate proteome for further analysis. Furthermore, recent study by Kliuchnikova *et al*. on characterization of AH proteome in cataract, glaucoma, and pseudoexfoliation syndrome was conducted sans the depletion steps due to the aforementioned reasons [13]. The comparison analysis of total number of AH proteins identified from other studies have already been discussed by Kim *et al*. and Kliuchnikova *et al*. [13, 17]. Generally, the total number of identified AH proteins were 71 [80], 54 [10], 355 [2], 137 [9], 81 [17], 198 [1], 445 [5], 242 [14], 269 [13], 763 [79] and as many as 819 [12]. The direct comparison of the 147 AH proteins identified from this study with previous study are challenging due to the different database search criteria, as described by Kliuchnikova *et al*. [13]. Nevertheless, in total 86% of the proteins identified in this study, representing the core proteome of AH, were also identified in previous studies as follows, 65% [2], 50% [1], 61% [5], 66% [79], 73% [13] and 78% [12] (complete data in Table F in S1 File). The characterization of AH proteins in this study also demonstrated that the identified proteins can be clustered into major biological processes, which included the defence, immune and inflammatory responses. A large number of these proteins were localized in the extracellular space and cytoplasm and possess numerous molecular functions.

Moreover, the label-free quantitative discovery proteomics strategy described in this study was instrumental to provide first evidence that gender-specific differences exist in the AH proteome. Therefore, it is highly recommended that studies, depending on their objectives, should divide their samples by gender to distinguish any specific alterations associated with sex difference. Since it is an established practice to collect AH during cataract surgeries, the proteome of these samples will differ from that of healthy persons in composition and concentration of some proteins [81, 82]. Label-free quantification has emerged as the simplest and most economical platform for in-depth analyses of differentially expressed/ abundant proteins with high precision [13, 83]. Furthermore, the development and improvement of the existing algorithms and software, such as the MaxQuant computational proteomics suite employed in the present study, have provided state-of-the-art quantification accuracy and coverage [37, 84]. On the contrary, protein quantification with isotopic labelling (e.g. iTRAQ) is also a commonly employed method owing to their quantitative accuracy, coverage and robustness [1, 5, 12]. Nevertheless, despite their usefulness, the labelling methods often require additional preparation steps, which increase the complexity of the analysis and consequently hamper detection of subtle yet crucial proteome changes, mostly the low abundant AH proteins.

In conclusion, this study is the first to reveal significant inter-gender variations in the differentially abundant proteins in human AH. These results are envisioned to be important reference points and are highly relevant for sample selection in future studies utilizing human AH in various pathological conditions.

## Supporting information

S1 File Table A in S1 File: General description and the total protein concentration yielded from the AH samples. Table B in S1 File: Complete list of the proteins identified from the AH samples employing the discovery proteomics strategy. Table C in S1 File: Summary of the statistical analysis of the proteins identified from the AH samples utilizing Perseus software. Table D in S1 File: Complete list of the over-represented GOBP terms of the AH proteins analysed employing the DAVID tool. Table E in S1 File: Complete lists of the PPI networks of the AH proteins analysed employing the Ingenuity Pathways Analysis software. Table F in S1 File: List of the proteins identified from the AH samples in the current study in comparison to previous studies in the literature.
(XLS)
